# Supplementary material for: A Likelihood Approach for Real-Time Calibration of Stochastic Compartmental Epidemic Models
Source: PLoS Comput Biol. 2017 Jan 17;13(1):e1005257. doi: 10.1371/journal.pcbi.1005257 (PMC5240920; doi:10.1371/journal.pcbi.1005257)
Supplement: S1 File — (TAR.GZ) [file pcbi.1005257.s014.tar.gz › HSPH_Online-SI-Revision/IPoi09/n1-severe/n1-severe_BENCH2_summary.pdf]

peak −8	3w spec	3w cum	5w spec	5w cum	end	3w spec LOG	3w cum LOG	5w spec LOG	5w cum LOG	end LOG
mean	162.5	163.1	103.3	122.1	44.3	0.7	0.6	0.7	0.7	0.6
median	103.5	131.8	87.7	96.6	43.3	0.6	0.6	0.6	0.6	0.4
quantile .1	62.1	72.3	40.	53.7	24.	0.3	0.3	0.3	0.3	0.1
quantile .9	280.4	272.7	163.1	206.	65.3	1.	1.	1.4	0.3	1.4
45	45	45	45	41						

peak −4	3w spec	3w cum	5w spec	5w cum	end	3w spec LOG	3w cum LOG	5w spec LOG	5w cum LOG	end LOG
mean	59.4	65.	58.4	49.9	38.5	0.3	0.3	0.4	0.3	0.2
median	48.3	58.2	49.2	41.9	34.5	0.3	0.3	0.2	0.2	0.2
quantile .1	27.6	30.1	28.1	23.3	24.	0.1	0.2	0.1	0.1	0.1
quantile .9	101.9	111.3	80.9	79.	59.7	0.4	0.4	0.9	0.1	0.5
44	45	38	41	29						

peak	3w spec	3w cum	5w spec	5w cum	end	3w spec LOG	3w cum LOG	5w spec LOG	5w cum LOG	end LOG
mean	64.4	53.1	75.8	52.8	53.1	0.2	0.2	0.3	0.2	0.2
median	49.9	45.	54.8	45.8	49.9	0.2	0.2	0.2	0.2	0.2
quantile .1	35.2	30.4	37.6	31.3	32.8	0.1	0.1	0.2	0.1	0.1
quantile .9	103.6	78.3	143.5	73.3	82.1	0.3	0.3	0.4	0.1	0.3
37	32	39	31	36						

peak +4	3w spec	3w cum	5w spec	5w cum	end	3w spec LOG	3w cum LOG	5w spec LOG	5w cum LOG	end LOG
mean	629.3	85.4	1298.6	95.9	114.2	0.3	0.2	0.5	0.3	0.3
median	65.4	54.8	72.9	59.2	58.3	0.2	0.2	0.3	0.2	0.2
quantile .1	38.8	37.6	46.6	38.7	43.3	0.2	0.2	0.2	0.2	0.2
quantile .9	187.3	140.9	555.7	145.	220.8	0.4	0.3	0.7	0.2	0.5
43	37	38	37	39						

peak +8	3w spec	3w cum	5w spec	5w cum	end	3w spec LOG	3w cum LOG	5w spec LOG	5w cum LOG	end LOG
mean	1119.	2243.7	884.4	2938.7	3726.6	0.5	0.4	0.6	0.5	0.5
median	76.6	64.2	78.1	66.1	67.9	0.3	0.3	0.5	0.3	0.3
quantile .1	47.	43.7	55.3	43.	46.5	0.2	0.2	0.3	0.2	0.2
quantile .9	4650.	301.2	3260.6	429.6	598.2	1.4	0.5	1.3	0.2	0.8
42	38	42	38	35	{}	{}	{}	{}	{}	{}
